# WHO calls on the global community to equalize the HIV response

**Published:** 2023-01

**1 December 2022** - On 1 December, World AIDS Day 2022, the World Health Organization (WHO) is calling on global leaders and citizens to boldly recognize and address the inequalities that are holding back progress in attaining the global goal to end AIDS by 2030.

WHO is joining global partners and communities in commemorating World AIDS Day 2022 under the theme “Equalize” - a message highlighting the need to ensure that essential HIV services reach those who are most at risk and in need, particularly children living with HIV, key populations to HIV and their partners.

“With global solidarity and bold leadership, we can make sure everyone receives the care they need,” said Dr Tedros Adhanom Ghebreyesus, WHO Director-General. “World AIDS Day is an opportunity to re-affirm and refocus on our shared commitment to end AIDS as a public health threat by 2030.”

HIV remains a major public health issue that affects millions of people worldwide. But our response is at risk of falling behind.

Of the 38 million people living with HIV, 5.9 million people who know they have HIV are not receiving treatment. A further 4 million people living with HIV have not yet been diagnosed. While 76% of adults overall were receiving antiretroviral treatment that help them lead normal and healthy lives, only 52% of children living with HIV were accessing this treatment globally in 2021. Approximately 70% of new HIV infections are among people who are marginalized and often criminalized. While transmission has declined overall in Africa, there has been no significant decline among men who have sex with men - a key population group - in the past 10 years.

**Overlapping epidemics of mpox and HIV**

Available WHO data show that among people confirmed to have mpox, a high number - 52% - were people living with HIV. Global data reported to WHO suggest that people living with mpox with untreated HIV appear to be at risk for more severe disease than people without HIV.

The current response to mpox shows that transmission can move quickly in sexual networks and within marginalized populations. But it can also be prevented with community-led responses and open attitudes to address stigma, and health and well-being can be improved and lives can be saved.

**Delivering for key populations of HIV**

This World AIDS Day, WHO recommends a renewed focus to implement WHO’s 2022 guidance to reach the HIV and related health needs of key populations and children.

“People must not be denied HIV services no matter who they are or where they live, if we are to achieve health for all,” said Dr Meg Doherty, WHO Director of the HIV, Hepatitis and STI programmes. “In order to end AIDS, we need to end new infections among children, end lack of treatment access to them, and end structural barriers and stigma and discrimination towards key populations in every country as soon as possible.”

With only eight years left before the 2030 goal of ending AIDS as a global health threat, WHO calls for global solidarity and bold leadership from all sectors to ensure we get back on track to ending AIDS and, with that, end new syndemics, such as the recent mpox global outbreak.

Available from: https://www.who.int/news/item/01-12-2022-who-calls-on-the-global-community-to-equalize-the-hiv-response

